# Is there really an accelerated phase of chronic myeloid leukaemia?

**DOI:** 10.1038/s41375-024-02316-5

**Published:** 2024-06-25

**Authors:** Robert Peter Gale, Qian Jiang, Jane F. Apperley, Andreas Hochhaus

**Affiliations:** 1 Centre for Haematology, Department of Immunology and Inflammation, Imperial College of Science, Technology and Medicine, London, UK; 2 Peking University People’s Hospital, Peking University Institute of Haematology National Clinical Research Center for Haematologic Disease, and Beijing Key Laboratory of Hematopoietic Stem Cell Transplantation, Beijing, China; 3 Klinik für Innere Medizin II, Universitätsklinikum Jena, Jena, Germany

**Keywords:** Cancer, Diseases

Naming something to distinguish it from other things is foundational. An example in chronic myeloid leukaemia (CML) is claiming some laboratory co-variates as define *accelerated* phase distinguishing it from *chronic* and *blast* phases. However, there should be convincing conceptual, biological and clinical bases for this distinction. In Genesis the 1^st^ thing God did after separating light from darkness was to name the light *day*. Adam was then tasked by God to name the animals. Fair enough. But what if Adam named the 1^st^ dove he saw a dove and a 2^nd^ dove, an eagle. Namely. giving something a name does not automatically make it a distinct entity.

These biblical anecdotes are relevant to the considerable recent controversy whether there is an *accelerated* phase of CML [1]. For example, the 2022 WHO classification of myeloid neoplasms deleted accelerated phase stating: *The designation of AP [accelerated phase] has thus become less relevant where resistance stemming from ABL1 kinase mutations and/or additional cytogenetic abnormalities and the development of BP [blast phase] represent key disease attributes. Accordingly, AP is omitted in the current classification in favor of an emphasis on high-risk features associated with CP progression and resistance to TKIs* [2]. In contrast, the 2020 European Leukemia Net (ELN) recommendations and the 2022 International Consensus Classification (ICC) retain accelerated phase [3, 4]. A recent 2024 National Comprehensive Cancer Network (NCCN) guidelines also retains accelerated phase [5]. The issue gets even more complicated in light of different criteria for accelerated phase [3, 6, 7].

Let’s consider data supporting the 2022 WHO position. Biologically, we think chronic phase CML is best considered a *preleukaemia* rather than a leukaemia. We suggest this because chronic phase cells differentiate normally, respond to normal physiological regulatory signals regulatory signals such as granulocyte- and granulocyte/macrophage colony stimulating factors (G- and G/M-CSFs), function mostly normally, have normal or near normal lifespans and undergo normal terminal differentiation. There are simply too many of them. There is no convincing evidence of uncontrolled proliferation in chronic phase CML. For example, the cells do not form myeloid colonies spontaneously in vitro and mostly function normally in vivo. In persons with chronic phase CML blood granulocyte concentration increases appropriately in infections and in rare persons with cyclical neutropenia and chronic phase CML the blood granulocyte concentration cycles like persons with cyclical neutropenia without CML. Lastly, in untreated chronic phase CML the blood granulocyte concentration usually reaches an stable apogee [8–10]. Moreover, the gene expression profile (GEP) of chronic phase CML cells resembles normal CD34-postive cells [11]. The increased granulocyte mass in chronic phase CML is explainable by as few as 2–4 extra late-stage cell divisions.

Clinically, CML was initially divided into *chronic* and *terminal* or *blast* phases [12]. Many of these people were in what is termed blast phase and had brief survivals. The proposition of an intermediate accelerated phase was based on identifying co-variates at diagnosis or during the disease course associated with an increased risk of transforming to blast phase. These co-variates were identified before the era of TKI-therapy and differ between classifications [3, 6, 7]. However, accelerated phase was removed from the 2022 World Health Organization (WHO) classification of CML because with TKI-therapy few people with chronic phase CML transform to blast phase.

In an analysis of > 2000 people with CML in either chronic or accelerated phases at diagnosis based on the 2020 ELN and 2022 ICC criteria we found most subjects classified as accelerated phase had outcomes like persons in chronic phase identified as intermediate- or high-risk cohorts classified by the European Treatment and Outcome Study (EUTOS) Long-Term Survival score (ELTS) risk classification [13]. The other 20 percent had some worse outcomes but only if they failed to meet the 2020 ELN TKI-therapy-response milestones. A recent study reported failing to meet these milestones before 1-year has little impact on survival [14]. Noteworthily, even if someone is designated to be in accelerated phase they receive the same TKI-therapy a someone in chronic phase.

These data imply the co-variates defining accelerated phase are predominately correlated with late response to TKI-therapy rather than a distinct phase of CML. In other cancers, including the acute and chronic leukaemias, we do not create a new disease phase based on adverse predictive or prognostic co-variates at diagnosis or evolving under therapy. Why should we in CML? When these co-variates emerge in someone originally in chronic phase we suggest they are simply no longer in chronic phase. An example is acquisition high-risk additional cytogenetic abnormalities [15]. Interestingly, 48 years ago Sakurai and colleagues reported similar additional cytogenetic abnormalities in 1 or a few CML cells at diagnosis when large numbers of metaphases were analyzed [16]. This is, of course, not surprising given the estimated latency of chronic phase CML from acquiring *BCR::ABL1* to diagnosis is about 10 years based on data from the A-bomb survivors [17–19]. The implication is a person has the genetic abnormality which causes CML years before the diagnosis with considerable time to develop additional cytogenetic abnormalities currently associated with accelerated phase. Lastly, GEP data suggest only 2 phases of CML [11]. Based on these biological and clinical considerations and data we suggest eliminating accelerated phase as was done in the 2022 WHO classification is appropriate.

Having eliminated accelerated phase the last issue is what to call the non-chronic phase of CML when it transitions from being a preleukaemia to a leukaemia? The original terms were *terminal* and *blast* phases [12, 20]. Acute and advanced phases were added later. Blast, leukaemia and acute phases are inaccurate descriptors as some people in this phase lack increased blasts or have only decreased platelets or increased basophils. Terminal, acute, advanced and *not chronic* phases are more accurate.

In summary, based on the biological and clinical considerations and data we cite we suggest CML has 2 phases, chronic and terminal, advanced or non-chronic phases. We favour advanced which gives latitude of interpretation. Regardless of which term is used we need consensus. Lastly, the data we cite support the 2022 WHO classification of CML eliminating accelerated as a phase of CML.
